# Preclinical atherosclerosis in adolescents with psychotic or bipolar disorders investigated with carotid high‐frequency ultrasound

**DOI:** 10.1002/brb3.1862

**Published:** 2020-09-30

**Authors:** Hannes Bohman, Ingrid Agartz, Shiva Mansouri, Tord Naessen, Mathias Lundberg

**Affiliations:** ^1^ Department of Neuroscience, Child and Adolescent Psychiatry Uppsala University Uppsala Sweden; ^2^ Department of Clinical Science and Education Södersjukhuset Karolinska Institutet Stockholm Sweden; ^3^ NORMENT and K.G. Jebsen Centre for Psychosis Research Institute of Clinical Medicine University of Oslo Oslo Norway; ^4^ Department of Psychiatric Research Diakonhjemmet Hospital Oslo Norway; ^5^ Department of Women's and Children's Health Uppsala University Uppsala Sweden

**Keywords:** adolescents, atherosclerosis, bipolar disorder, psychosis, ultrasound

## Abstract

**Objective:**

Early‐onset psychosis (EOP) and bipolar disorder (EOBP) (at <18 years of age), are associated with an increased future risk of cardiovascular disease (CVD) and premature death. Yet it is unknown whether the arteries show visible signs of atherosclerosis in EOP and EOBP. This study investigated whether having EOP or EOBP was associated with detectable signs of preclinical atherosclerosis.

**Method:**

By using 22 MHz high‐frequency ultrasound, different layers of the arterial wall of the left common carotid artery (LCCA) were assessed in 77 individuals with EOP (*n* = 25), EOBP (*n* = 22), and in age‐matched healthy controls (*n* = 30). Conventional CVD confounders were included in the analyses.

**Results:**

Adolescents with EOP and EOBP, compared to controls, had a significantly thicker LCCA intima thickness (0.132 vs. 0.095 mm, *p* < .001) and intima/media ratio (0.24 vs. 0.17 *p* < .001). There was a nonsignificant intima difference between EOP and EOBP. Conventional CVD risk factors did not explain the association between EOP/EOBP and intima thickness. In the group of EOP/EOBP, there was a significant correlation between the dose of current antipsychotic medication and intima thickness; however, the correlation was attenuated to a nonsignificant level when adjusted for global function.

**Conclusions:**

Adolescents with EOP or EOBP had an increased LCCA intima thickness, interpreted as a sign of preclinical atherosclerosis. Global function of the disorders was the strongest determinant of intima thickness. The findings, if replicated, might have implications for long‐term treatment of EOP and EOBP in order to reduce a future risk of CVD.

## Graphical abstract

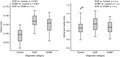

## Significant Outcomes

- Adolescents with EOP and EOBP had a significantly thicker LCCA intima thickness compared to age‐matched healthy controls, interpreted as a sign of preclinical atherosclerosis.
- The increased intima thickness was explained neither by common cardiovascular risk factors, nor by antipsychotic medication.
- Global function that combines symptom severity and low functioning was the strongest determinant of increased intima thickness.

## Limitations

- Some cardiovascular risk markers measured in serum/plasma were collected from the medical records for the patients with EOP and EOPD and were not available for the healthy controls.
- The controls were matched for age, but there was an over‐representation of females among the controls which somewhat limits the comparability between the groups
- We did lack information about the CVD risk marker smoking.

## 1 INTRODUCTION

Psychotic disorders, including, for example, schizophrenia or schizoaffective disorder, are devasting disorder with a prevalence of about 0.5% (Moreno‐Küstner et al., 2018). The psychotic disorders affect the ability to experience adequate emotions, think clearly, and participate in social life. In fact, even when most of the subjects are responsive to treatment less than a third receive recovery (Weinberger & Harrison, 2011). Bipolar disorder is as well among the most severe mental disorders and has a prevalence of about 1.4% (Merikangas et al., 2007). Bipolar disorder is characterized by episodic depression and mania/hypomania. Compared to psychotic disorders, the rate of remission and recovery is better, and pharmacological treatment is less dependent on second‐generation antipsychotics (SGA) with its metabolic side effects.

Individuals with psychosis and bipolar disorder have an increased risk for cardiovascular disease (CVD), compared with the general population. Moreover, the life expectancy is considerably shortened with about 11–22 years and most of the years are lost due to CVD (Tiihonen et al., 2009). Several important risk factors have been observed in this group, for example, high levels of cholesterol and low‐density lipids, obesity, smoking, less exercise, and unhealthy diet (Davidson et al., 2001; Mucheru et al., 2018). Research over several decades have shown a connection between pharmacological therapy for psychotic and bipolar disorders and development of risk markers for CVD (Correll et al., 2009; Newcomer, 2006; Taylor & Macqueen, 2006). In the conditions of psychotic and bipolar disorders, the second‐generation antipsychotics (SGA), that are often used due to their efficacy and low frequency of extrapyramidal side effects, have been associated with weight gain and they have a negative impact on glucose and lipid metabolism (Correll et al., 2009). SGA is believed to contribute to the increased risk of CVD associated with these disorders (Reynolds & Kirk, 2010). For children and adolescents, this is of great concern since they are especially sensitive for pharmacological metabolic side effects (De Hert et al., 2011).

Yet other pathological effects on the blood vessels than pharmacological treatment with SGA and conventional risk factors could be of importance for the early development of CVD. Already before the introduction to psychopharmacological treatment, an increased risk of developing CVD among patients with psychotic disorders has been observed (Greenhalgh et al., 2017). While the risk in the general population of developing cardiovascular disease has fallen significantly in recent years in developed countries (Berg et al., 2014; Björck et al., 2009) no such decline have not been observed in individuals with psychosis and bipolar disorders (Lawrence et al., 2013). Studies indicate that the decline in the general population of cardiovascular disease is strongly correlated with the use of functional CVD risk factor algorithms, in the primary care, with good efficiency in identifying patients with risk of developing CVD diseases (Karjalainen et al., 2017; Piepoli et al., 2016). However, recent studies indicate that currently used CVD risk factor algorithms may not be functional for identification of CVD risk patients within population with psychotic disorders (McLean et al., 2014). In the study of McLean et al, individuals with schizophrenia developed CVD considerably earlier than the general population, at average age 55 years. Yet, only a small percentage (3.2% of men and 7.5% of women), under age of 55 with CVD, were correctly identified as high‐risk individuals for CVD (McLean et al., 2014) according to the JBS (Joint British Societies score) risk algorithm (JBS3 Board Joint British Societies, 2014). Conventional cardiovascular risk factors only partly seem to explain the increased incidence of CVD in younger individuals with psychotic and bipolar disorders.

Based on these findings, a specific CVD risk algorithm may be needed for patients with psychosis and bipolar disorder that includes alternative CVD biomarkers to identify the CVD risk in younger individuals with psychosis and bipolar disorders.

Furthermore, children's and adolescents' mental disorders such as EOBP have been identified by an international expert panel as risk conditions associated with accelerated atherosclerosis and development of early CVD, and it has been suggested that there is a substantial window of opportunity to intervene to prevent these outcomes among children and adolescents (Goldstein et al., 2015). Thus, it is of great importance to determine whether or when the early signs of atherosclerosis appear in EOP and EOBP, and how potential early changes are related to putative CVD risk factors.

### 1.1 Aims of the study

In this study, we aimed to investigate whether ultrasonographic signs of preclinical atherosclerosis are evident in adolescents with EOP or EOBP, using 22 MHz high‐frequency ultrasound (HFU). Furthermore, we investigated whether the degree of any carotid arterial affection in the group of adolescents with EOP and EOBP was associated with conventional risk factors for CVD and antipsychotic medication. We hypothesized that children and adolescents with EOP and EOBP show signs of preclinical atherosclerosis measured with HFU (22 MHz) and that signs of preclinical atherosclerosis would not be explained by conventional risk markers or antipsychotic medication.

## 2 MATERIAL AND METHODS

### 2.1 Study population and procedure

Patients were recruited from the specialist care unit of psychosis and bipolar disorder in the department of Child and Adolescent Psychiatry unit, Stockholm County Health Care Area. The Stockholm county consists of 430,000 children up to 18 years and the unit investigates for diagnosis and treat children and adolescents with EOP and EOBP referred from local Child and Adolescent Psychiatry Clinics for specialist evaluation and treatment. About 120 patients are currently treated in the unit and there are about 30 new patients yearly. Patients are recruited with referral from either the child or adolescent psychiatry in‐patient or outpatient care.

All patients referred for investigation in the specialist unit are investigated in 3 separate sessions by any of 4 psychiatric specialists working in the unit. The investigation includes self‐rating forms for psychotic and bipolar disorder and differential diagnoses; the Development and Well‐Being Assessment (Goodman et al., 2000), Psychotic‐Like Symptom Screener (Kelleher et al., 2011), Child Mania Rating Scale (Pavuluri et al., 2006), the Mood Disorder Questionnaire (Hirschfeld et al., 2000), and the Adolescent Dissociative Experiencing Scale (Armstrong et al., 1997). Individual anamnestic interviews with the child and the parents are performed concerning symptoms of the disorders. Information from other informants is collected during the assessment. If the patient is considered to fulfill the criteria for either psychotic disorder or bipolar disorder, the patient is enrolled in the unit. Patients who fulfilled diagnosis for psychotic disorder between the years 2014 and 2019 were asked together with their parents if they wanted to be included in a multi‐center study, where Stockholm Child and Adolescent Psychosis Study SCAPS is a part (Smelror et al., 2018). For the present study, adolescents with bipolar disorder were also added. Before being included in the study, a round table discussion between two senior child and adolescent psychiatrists decided the diagnoses according DSM‐5. Psychotic disorder diagnoses included schizophrenia, schizoaffective disorder, psychotic depression, and unspecified psychosis. Bipolar disorder included bipolar 1 and 2 disorders without psychosis. Patients with known cardiac disease were ruled out. Patients who were very low functioning or who displayed severe symptoms were not included due to difficulties to give consent or participate in the investigations. For this study, 25 adolescents with psychotic disorder and 22 adolescents with bipolar disorder were recruited with an average age of 16.7 years.

A healthy control group of 30 adolescents of the same ages who were without experience of child and adolescent mental health care and who had no evidence of cardiac disease were recruited to undergo the same investigation and measurements during the same time period. The average age of the controls was 17.1 years.

### 2.2 Assessments

At the time of admission and ultrasound investigation was performed, the patients were investigated for blood pressure, pulse rate, height, weight, body mass index (kg/m^2^), waist/hip ratio, blood samples, and physical fitness. Blood pressure was measured after about 15 min rest in supine position, on the right upper arm, with an automated blood pressure equipment, Umed ico 12 × 35 cm or a size appropriate for the arm circumference. Venous blood was collected. Serum and plasma were saved at −80°C for laboratory analysis. From the medical records, values on routine laboratory analyses were collected. Serum/plasma samples included cholesterol, low‐density lipids (LDL), high‐density lipids (HDL), thyroid‐stimulating hormone (TSH), S‐Insulin, erythrocyte sedimentation rate (ESR), and hemoglobin (Hb). Level of training more than 30 min was measured with a self‐rating scale: never = 0, once a week = 1, twice a week = 2, and several times a week = 3. Functioning and symptom severity of the disorder was measured with Children's Global Assessment Scale (CGAS) (Smelror et al., 2018), where 100 points was the highest function possible and 1 was the lowest function possible. Data on antipsychotic medication were collected. There were 31 adolescents (19 with EOP and 12 with EBPD) who were currently treated with antipsychotic medication, most of them with SGA, for example, risperidone, aripiprazole, olanzapine or, quetiapine. A continuous variable with equivalent doses was used; for example, 1 mg haloperidol corresponded to 1.7 mg olanzapine, to 0.5 mg risperidone, and to 2.5 mg aripiprazole (Kroken et al., 2009).

### 2.3 High‐frequency ultrasound (HFU) and carotid intima‐media thickness (cIMT) of the artery wall

The gold standard method, noninvasive ultrasound (CCA‐IMT), measures carotid intima‐media thickness (cIMT). In CCA‐IMT, the combined inner blood vessel layers, intima and media thickness, are measured. The increasing thickness of the media and intima layers combined is interpreted a sign of atherosclerosis. It is a method to uncover atherosclerosis and is a valid method to verify CVD in clinical populations (Carpenter et al., 2016). In adults, cIMT is predictive of future cardiovascular events including stroke and myocardial infarction (O'Leary et al., 1999) It has also been the case for patients with chronic inflammatory diseases such as rheumatoid arthritis, in whom cIMT predicted the risk of cardiovascular events (Gonzalez‐Juanatey et al., 2009). In children, increased cIMT has been found in obesity, hypertension, diabetes mellitus, and metabolic syndrome (Urbina et al., 2009).

However, with advancing age/atherosclerosis, the thickness of the intima layer increases, whereas the media layer decreases (Gussenhoven et al., 1991). These differential changes might in some conditions limit the usefulness of conventional cIMT at younger ages because a thinner media layer might conceal the initial slightly increasing thickness of the intima layer. cIMT has in some studies showed limited sensitivity in detecting early changes in the young population <18 years (Polak & O'Leary, 2016). HFU, that measure intima and media separately have, on the other hand in some studies of younger individuals been able to detect early signs of atherosclerosis where the cIMT method have not (Akhter et al., 2014; Bohman et al., 2010; Leonard et al., 2011; Ljunggren et al., 2018). For this reason, we chose to use HFU which is able to measure the media and intima layers separately.

To assess the thickness of the individual wall layers in the left common carotid artery (LCCA), we used a high‐resolution ultrasound equipment (Osteoson® Minhorst GmbH, Meudt, Germany) fitted with a broadband probe with 22 MHz center frequency.

The point of maximal pulsation in front of the sternocleidomastoid muscle with the subjects sitting in 45‐degree position and looking straight ahead after a period of 15 min rest was examined. The probe was applied perpendicular to the length of the artery and until a three‐layered image was obtained, with two echo dense zones (adventitia and intima) with an echo lucent area (media) between them, followed by an echo lucent artery lumen. Each image provided a point estimate, and about 10‐point estimates were saved for each subject. Measurements of the thickness of the individual arterial layers were performed off‐line and blindly by 2 assessors. The examiner strived to measure at the points where the contrast of the intima and media was highest. Means of the about 10 measurements were calculated and compared. The average result of the measurements between the two assessors was used in for calculation in the analysis. The consistency between the assessors was satisfactory. The intima measurements deviated less than 0.015 mm among the assessors from the calculated average in 82% of the individuals and deviated less than 0.02 mm from the average in 95% of the individuals.

### 2.4 Statistical analysis

*T* test was used to compare mean values. The variables investigated were normally distributed with acceptable variance. Since some of the groups that were compared were small or differed in size, nonparametric tests were also performed. However, the results did not differ from the *t* test and are therefore not shown in the tables. Spearman's rank correlation was used to calculate correlations. To adjust for potential confounders, we conducted a binary linear regression models in the whole sample. Linear regression analyses were also conducted in the combined EOP and EOBP group. In the regressions, all individuals, including those without antipsychotic medication (*n* = 16), were included not to lose power. The individuals without antipsychotic treatment were assigned the value of 0. A few individuals lacked values for hip/waist (*n* = 3) and were not included in the analyses. All statistical analyses were performed in IBM SPSS Statistics for Windows, version 24.

### 2.5 Ethics

After a complete description of the study, written informed consent was obtained from the participants and from one of their parents. If the patient was younger than 16 years old, both parents gave their consent. The study was approved by the local ethical vetting board of Karolinska Institutet, Stockholm, Sweden.

## 3 RESULTS

### 3.1 Cardiovascular risk factors

Descriptive differences of CVD risk factors in male and female adolescents with EOP and EOBP, compared with healthy controls, are presented in Table 1. Altogether, adolescents with EOP/EOBP combined, compared to healthy controls, had significantly higher values for diastolic pressure (*p* < .05), waist circumference (*p* < .01), waist/hip ratio (*p* < .01), BMI (*p* < .05), intima (*p* < .001), and intima/media ratio (*p* < .001) compared to the controls. Men with EOP or EOBP had significantly higher waist/hip ratio (*p* < .001), intima (*p* < .001), and intima/media thickness ratio (*p* < .01). There were more men in the combined group of EOP and EOBP and more women among the healthy controls (*p* < .05). Females with EOP and EOBP had significantly higher diastolic blood pressure (*p* < .05), higher pulse rate (*p* < .05), waist circumference (*p* < .05), waist/hip ratio (*p* < .01), BMI (*p* < .05), intima thickness (*p* < .001), and intima/media thickness ratio (*p* < .001). In contrast, conventional cIMT, which measures the sum of the media and intima thickness, was not significantly different between the study groups, neither for men nor for women. Nonparametric calculation test with Mann–Whitney was also performed since in some calculations the numbers were small. The results did not differ from the *t* test results except for pulse frequency in women. The significant result (*p* = .037) turned borderline significant (*p* = .074). Data are not presented in table.

**TABLE 1:** Descriptive table of CVD risk factors in male and female adolescents with early‐onset psychosis or early‐onset bipolar disorder compared to healthy controls

	All			Males			Females		
	Probands	Controls	*p* 95%	Probands	Controls	*p* 95%	Probands	Controls	*p* 95%
*n*	47	30		25	8		22	22	
Age	16.7	17.1	n.s	17.5	17.1	n.s	16.3	17	n.s
Sex (M/F)	(25/22)	(8/22)	**<.05**						
Systolic blood pressure (mm Hg)	118	114	n.s	118	121	n.s	117	112	n.s
Diastolic blood pressure (mm Hg)	74	69	**<.05**	73	72	n.s	75	68	**<.05**
Pulse rate (beat/min)	78	70	**<.05**	73	75	n.s	83	68	**<.05**
Waist circumference (cm)	82	74	**<.01**	87	78	n.s	76	72	n.s
Hip circumference (cm)	91	94	n.s	94	97	n.s	88	93	n.s
Waist/hip ratio	0.90	0.79	**<.001**	0.93	0.82	**<.001**	0.87	0.78	**<.01**
Height (cm)	172	168	n.s	177	173	n.s	166	167	n.s
Weight (kg)	75	63	**<.01**	82	71	n.s	67	61	n.s
BMI	25	22	**<.05**	26	24	n.s	24	22	n.s
Common carotid artery									
Intima thickness	0.132	0.095	**<.001**	0.133	0.101	**<.001**	0.130	0.093	**<.001**
Media thickness	0.594	0.587	n.s	0.663	0.725	n.s	0.515	0.537	n.s
Intima/med ratio	0.24	0.17	**<.001**	0.21	0.14	**<.01**	0.27	0.18	**<.001**
Intima‐media thickness	0.735	0.682	n.s	0.796	0.825	n.s	0.645	0.630	

Note

Differences in mean values.

Abbreviations: BMI, Body Mass Index; *p*, significance.

Bold p‐values indicate statistically significant differences.

### 3.2 Differences in intima thickness between EOP versus EOBP versus controls

**FIGURE 1:**
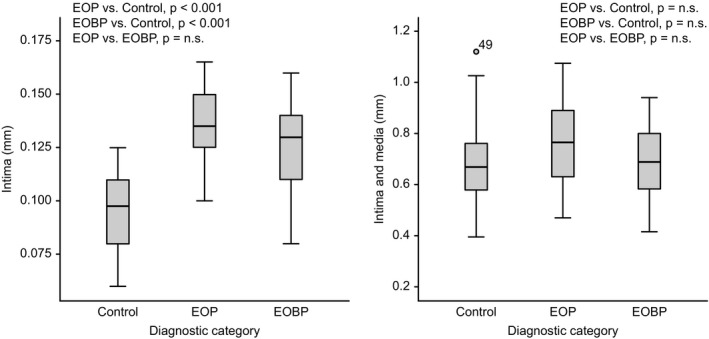
Box plot with median and upper and lower quartile of intima thickness (mm) in adolescents with early‐onset psychosis (*n* = 25), early‐onset bipolar disorder (*n* = 22), and healthy controls (*n* = 30) compared

In Figure 1, we demonstrate differences in intima thickness between adolescents with the EOP and EOBP groups and the healthy controls. The intima thickness was borderline significant between EOP and EOBP (0.137 mm vs. 0 0.126 mm, *p* = .056), whereas EOP (*p* < .001) and EOBP (*p* < .001) independently differed significantly from healthy controls (0.095 mm).

In the figure, the sum of intima and media thickness, which is used in cIMT, is shown. EOP or EOBP did not differ significantly from healthy controls. Patients with EOP did not differ from patients with EOPD.

There were some differences in CVD risk factors between EOP and EOBP. CGAS differed significantly between EOP and EOBP (45 vs. 62 points, *p* < .001). The use of antipsychotic medication, measured in dose equivalents, differed significantly between EOP and EOBP (4 mg vs. 1 mg, *p* < .001). No other variables investigated differed significantly.

### 3.3 Linear regression of cardiovascular risk factors analyses in the pooled sample

To analyze which CVD risk factors that strongest correlated with the early signs of atherosclerosis, measured as intima thickness, a regression analysis was performed in the pooled sample. The analysis included a diagnosis of EOP and EOBP combined (EOP/EOBP) sex, pulse rate, diastolic blood pressure, waist/hip ratio, and BMI (Table 2). In the bivariate analysis, waist/hip ratio (*p* < .05), sex (*p* < .05), and diagnoses of EOP/EOBP (*p* < .001) significantly correlated with intima thickness. In the multivariate analyses, only EOP/EOBP was significantly correlated with intima thickness (*p* < .001). Analyses were also performed with intima/media ratio, but they did not differ from the calculations conducted with intima only.

**TABLE 2:** Linear regression analyses of adolescents with common carotid artery intima thickness and different cardiovascular risk factors in a pooled sample with early‐onset psychosis, early‐onset bipolar disorder, and controls

Intima thickness	Bivariate		Multivariate	
	Coef.	*p*	Coef.	*p*
EOP/EOBP	0.033	**<.001**	0.035	**<.001**
Females	0.014	**<.05**	0.007	n.s
Waist/hip ratio	0.085	**<.05**	0.025	n.s
BMI	0.001	n.s	0.001	n.s
DBP	0.000	n.s	0.000	n.s
SBP	0.000	n.s	0.000	n.s
Pulse	0.000	n.s	0.000	n.s
			*R* ^2^ = 0.445	

Note

Reference category = controls. *N* = 77.

Abbreviations: BMI, Body Mass Index; Coef., unadjusted beta coefficient *p* = significance level; DPD, diastolic blood pressure; EOD/EOBP, Early‐Onset Psychosis/Early‐Onset Bipolar disorder; SBP, systolic blood pressure.

Bold p‐values indicate statistically significant differences.

### 3.4 Rank correlations of significant CVD risk factors

In adolescents with EOP/EOBP, cardiovascular risk factors were analyzed separately. Analyses with Spearman's rank correlation are shown in Table 3. Antipsychotic medication equivalent dose (*r_s_* = 0.36; *p* < .05) and s‐Insulin (*r_s_* = −0.37; *p* < .05) positively correlated with intima thickness. Symptom/functioning (*r_s_* = −0.47; *p* < .01), measured with CGAS, was negatively correlated with intima thickness.

**TABLE 3:** Association between intima thickness in common carotid artery and different risk factors for cardiovascular disease in early‐onset psychosis and early‐onset bipolar disorder

	Correlation coefficient *r_s_*	*p*
APM equivalent	**0.36**	**.020**
CGAS	**−0.47**	**.001**
TG	0.09	n.s
Cholesterol	0.03	n.s
HDL	0.15	n.s
LDL	−0.15	n.s
HDL/LDL	−0.16	n.s
S‐Insulin	**0.37**	**.041**
ESR	−0.10	n.s
DBP	0.05	n.s
SBP	−0.22	n.s
Pulse rate	0.05	n.s
Waist/hip ratio	0.21	n.s
BMI	−0.02	n.s

Note

*N* = 47. Spearman's rank correlation.

Abbreviations: APM equivalent, Antipsychotic medicine equivalent; BMI, body mass index; CGAS, Children's Global Assessment Scale; DPD, Diastolic Blood Pressure; ESR, Erythrocyte Sedimentation Rate; HDL, High‐Density Lipids; LDL, Low‐Density Lipids; SBP, Systolic Blood Pressure; TG, Tri Glyceride.

Bold p‐values indicate statistically significant differences.

### 3.5 Stepwise regression analyses of significant CVD risk factors

Finally, stepwise regression analyses were performed to analyze which risk factor best explained the association with intima thickness and cardiovascular risk factors in EOP and EOBP. The analysis included sex, waist/hip ratio, antipsychotic medication dose, and CGAS. When antipsychotic medication dose was added to sex and waist/hip ratio, antipsychotic medication dose was significantly associated with intima thickness, (*p* < .01), while sex and waist/hip ratio were not. When global function measured with CGAS was added, CGAS negatively correlated with intima thickness (*p* < .05) but the dose of antipsychotic medication did not.

The variables s‐insulin (*n* = 28) that were correlated with intima thickness are not shown in Table 4. The low number individuals with recorded S‐Insulin made the variable not suitable for the analyses.

**TABLE 4:** Linear regression analyses of intima thickness in common carotid artery and different risk factors for cardiovascular disease in adolescents with early‐onset psychosis and early‐onset bipolar disorder

	Model 1		Model 2		Model 3		Model 4	
	Coef.	*p*	Coef.	*p*	Coef.	*p*	Coef.	*p*
Gender	−0.014	**.024**	−0.004	n.s	−0.002	n.s	−0.001	n.s
Waist/hip ratio			0.077	**.042**	0.036	n.s	0.048	n.s
APM equivalent					−0.004	**.002**	−0.003	n.s
CGAS							0.003	**.040**

Note

*N* = 44.

Abbreviations: APM equivalent, Antipsychotic medicine equivalent; CGAS, Children's Global Assessment Scale; Coef., beta coefficient.

Bold p‐values indicate statistically significant differences.

## 4 DISCUSSION

The main finding of this study was that adolescents with EOP or EOBP, when measured with HFU, had significantly increased thickness of the LCCA‐intima and higher intima/media thickness ratio, compared to healthy controls. The substantially thicker intima in both EOP and EOBP can be interpreted as signs of preclinical atherosclerosis and is an indication of already initiated pathology of the arteries in EOP and EOBP.

To our knowledge, this is the first study that has investigated EOP or EOBP with HFU. Only another study has used the method of ultrasound (cIMT) in youth with EOP or EOBP to our knowledge (Hatch et al., 2015). This study, that did not investigate differences between EOBP and healthy controls, found that intima and media thickness correlated with some CVD risk factors, which is in line with the present study.

In the present study, the choice of HFU method enabled us detect the differences between the groups. As seen in the figure, a significant difference with about 40% thicker intima compared to the controls was detected with HFU, while the cIMT did not show significant differences between EOP/EOBP and controls. The findings, if replicated, indicate that it might be possible to study signs of preclinical atherosclerosis already in EOP and EOBP using the HFU methods, and thus potentially assisting in the management of active measures to reduce their future risk of CVD.

Adolescent patients with EOP and EOBP also showed adverse values of other cardiovascular risk markers compared with healthy controls, such as higher blood pressure, waist/hip ratio, weight, BMI, and pulse rate. The findings are in line with other studies that have shown that CVD risk markers are elevated in this group of patients (Correll et al., 2017). CVD risk factors thus seem to develop early in life in individuals with psychotic and bipolar disorder, now also documented with regard to carotid artery wall morphology, indicating signs of preclinical atherosclerosis.

Earlier research has shown that metabolic risk factors in adolescents, like adiposity, and diabetes mellitus have been associated with increased cIMT thickness (Gooty et al., 2018; Park et al., 2015). Because CVD risk factors are believed to precede and to be involved in the development of atherosclerosis, we analyzed whether the CCA intima thickness could be explained by conventional CVD risk factors present in a pooled sample. However, when adjusted for CVD risk factors, having the diagnosis of EOP and EOBP combined was the only variable that significantly correlated with the intima thickening. Being diagnosed as EOP or EOBP might thus have an independent adverse effect on the artery wall, preceding or paralleling the development of other cardiovascular risk markers in this group of patients. The pathway for the development of CVD in patients with mental disorders might thus differ from that in the general population as proposed by some authors (McLean et al., 2014).

Because the initiation of antipsychotic medication is known to cause increased levels of other CVD risk markers (De Hert et al., 2011), the difference in intima thickness between a normal population and EOP and EOBP could possibly involve either an indirect or a direct effect of antipsychotic medication on the arteries. Therefore, correlation analyses were performed to assess the effect of antipsychotic medication and other cardiovascular risk factors on the arteries measured with HFU. The analyses showed that intima thickness significantly correlated positively with the equivalent dose of antipsychotic medication; however, among the different risk factors that were associated with increased intima thickness, only CGAS remained significant after mutual adjustment. The results of the analyses can be interpreted as that the global function that includes symptom severity and function of EOP and EOBP, measured as CGAS, is the strongest risk factor for preclinical atherosclerosis in younger individuals.

If the results from our study are confirmed, that is, that antipsychotic medicine is not associated with preclinical atherosclerosis when adjusted for confounders, the use of sufficiently high doses of antipsychotic medication, rather than to prioritize treatment with the lowest possible dose (and still accept some symptoms) would be preferable. Recent studies have shown that moderate doses of antipsychotic medicine, compared to none or low dose, lower the risk for future CVD and mortality (Torniainen et al., 2014). From this clinical point of view, more studies are needed to further analyze the effect of antipsychotic medicine on the arteries and the future risk of CVD.

Inflammatory processes may play an important role in the development of CVD in individuals with EOP and BPD. In adult patients with severe psychiatric disorders, development of atherosclerosis has been shown to involve inflammatory pathways and studies have shown an association between the level of plasma biomarker CRP and risk of cardiovascular disease (CVD) (Miller et al., 2014; Sicras‐Mainar et al., 2013). Moreover, recent studies have also indicated that young patients (<18 years), with psychiatric disorders, may also have increased levels of CRP (Rojas et al., 2011). In this study, only ESR was available as inflammatory marker and it was not associated with intima thickness. Further prospective studies with additional inflammatory markers are needed to investigate this possible association.

Furthermore, this cross‐sectional study cannot answer questions about causality between intima thickness and EOP/EOBP. It is possible that pathophysiological processes like inflammation results from the diseases and subsequently cause morphological changes of the blood vessels. But the opposite could hypothetically also be possible that an increased intima/endothelia thickness resulted by stress trigger and entertain the mental disorders. Some research suggests that schizophrenia may be a vascular‐ischemic disorder with endothelial pathology and defect vascular repair as important parts in the disorder (Hanson & Gottesman, 2005; Moises et al., 2015)

### 4.1 Limitations

Some limitations should be mentioned. This is a small‐scale study and performed on a clinical sample of patients; thus, there might be a risk of accidental findings and bias. To avoid risk of assessor bias, all ultrasound measurements were examined blinded and off‐line by two different assessors. However, the ultrasound investigation was performed nonblinded to the ultrasonographers, although information about the patient data was blinded. The fact that intima thickness correlated significantly with blinded information, such as CGAS, insulin resistance, and antipsychotic medication, should imply that the risk of such a bias is limited. The cardiovascular risk markers measured in serum/plasma were collected from the medical records for the patients with EOP and EOPD and were not available for the healthy controls. This limited the comparison between patients with EOP and EOPD and controls concerning risk factors of CVD, and some of the calculations could be performed in EOP and EOPD only. The controls were matched for age, but there was an over‐representation of females among the controls which somewhat limits the comparability between the groups. To cope with this problem, the results were presented separately for males and females and in addition sex were used as a confounder in calculations, when appropriate. The severity of the disease was measured with aid of CGAS. This measurement focuses on both decreased function and symptom severity but lacks some detailed information about symptoms. An instrument with more information about symptom severity might have added valuable information. Further, we did not include information about smoking. This information could have been valuable although the patients were young.

In conclusion, adolescents with EOP and EOBP had an increased CCA intima thickness and intima/media ratio, assessed by noninvasive high‐frequency ultrasound. This finding was interpreted as signs of preclinical atherosclerosis. The preclinical atherosclerosis was explained neither by common cardiovascular risk factors, nor by antipsychotic medication. Symptom severity or low functioning of the disorders was the strongest determinant. The findings, if replicated, might have implications for long‐term treatment of children and adolescents with EOP and EOBP and potentially assist in creating prevention strategies to reduce the future risk of cardiovascular disease associated with these disorders.

## CONFLICT OF INTEREST

The authors report no potential conflicts of interest.

## AUTHOR CONTRIBUTIONS

H.B. and M.L. contributed to the design of the study. H.B. collected data, completed data analysis and the writing of the paper. T.N, I.A. and S.M. participated in analyzing and interpreting the results and contributed to the writing of the paper.

### Peer Review

The peer review history for this article is available at https://publons.com/publon/10.1002/brb3.1862.

## Data Availability

The data are not publicly available due to ethical restrictions.
